# Diet restriction‐induced healthy aging is mediated through the immune signaling component ZIP‐2 in *Caenorhabditis elegans*

**DOI:** 10.1111/acel.12982

**Published:** 2019-06-18

**Authors:** Jeong‐Hoon Hahm, ChoLong Jeong, Hong Gil Nam

**Affiliations:** ^1^ Center for Plant Aging Research Institute for Basic Science Daegu Korea; ^2^ Department of New Biology Daegu Gyeongbuk Institute of Science & Technology (DGIST) Daegu Korea

**Keywords:** *C. elegans*, dietary restriction, longevity, mitochondria, TOR/S6K, ZIP‐2

## Abstract

Dietary restriction (DR) robustly delays the aging process in all animals tested so far. DR slows aging by negatively regulating the target of rapamycin (TOR) and S6 kinase (S6K) signaling pathway and thus inhibiting translation. Translation inhibition in *C. elegans* is known to activate the innate immune signal ZIP‐2. Here, we show that ZIP‐2 is activated in response to DR and in feeding‐defective *eat‐2* mutants. Importantly, ZIP‐2 contributes to the improvements in longevity and healthy aging, including mitochondrial integrity and physical ability, mediated by DR in *C. elegans*. We further show that ZIP‐2 is activated upon inhibition of TOR/S6K signaling. However, DR‐mediated activation of ZIP‐2 does not require the TOR/S6K effector PHA‐4/FOXA. Furthermore, *zip‐2* was not activated or required for longevity in *daf‐2* mutants, which mimic a low nutrition status. Thus, DR appears to activate ZIP‐2 independently of PHA‐4/FOXA and DAF‐2. The link between DR, aging, and immune activation provides practical insight into the DR‐induced benefits on health span and longevity.

## 1 INTRODUCTION, RESULTS, DISCUSSION

Dietary restriction (DR) without malnutrition effectively and reproducibly delays the age‐related decline in physiological functions in many organisms, including *C. elegans* (Walker, Houthoofd, Vanfleteren, & Gems, 2005). A key mechanism underlying the beneficial effects of DR is translational inhibition (Hansen et al., 2007). In *C. elegans*, translational inhibition induces the protective immune signal ZIP‐2, a bZIP transcription factor (Dunbar, Yan, Balla, Smelkinson, & Troemel, 2012).

**Figure 1:**
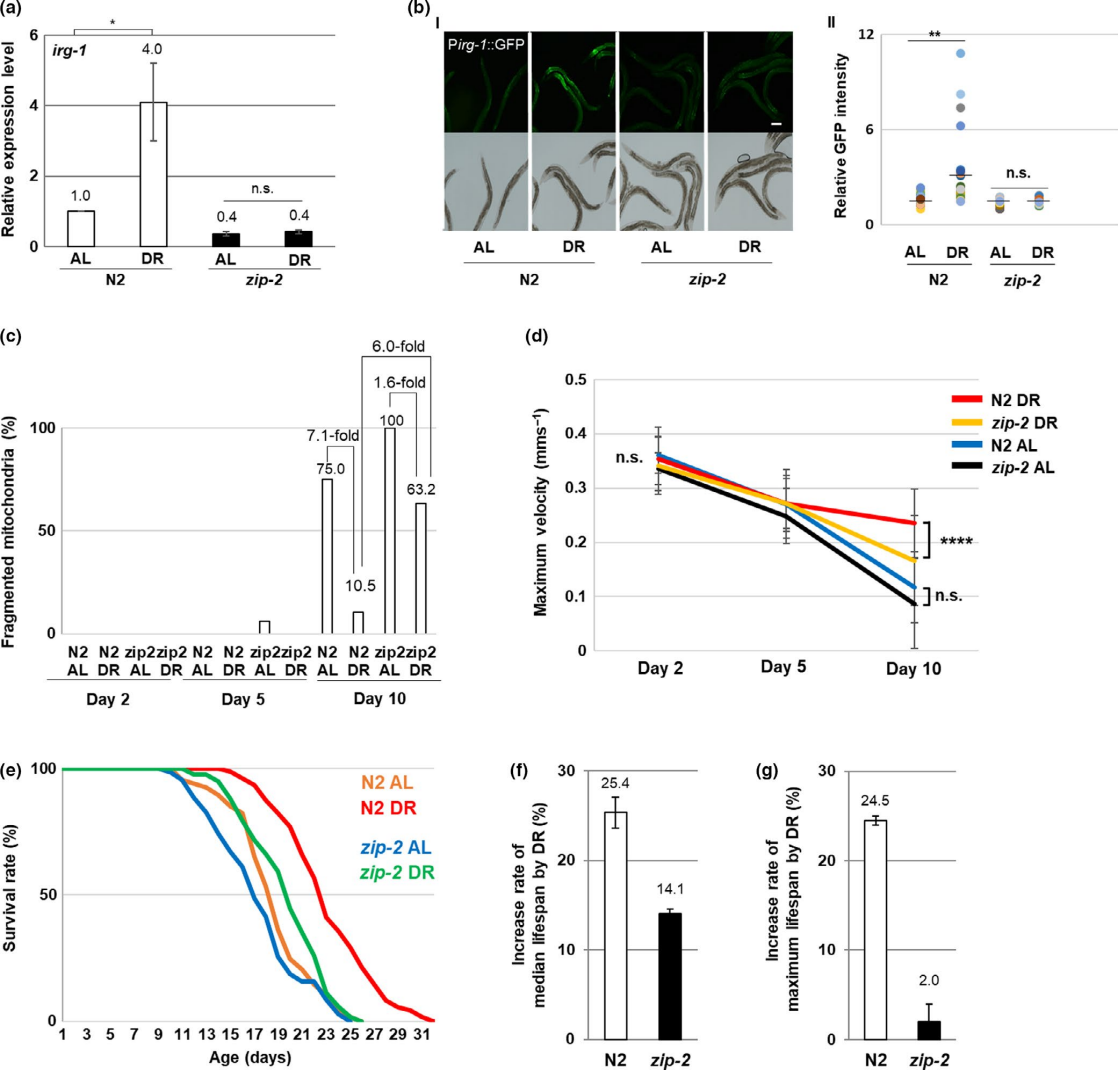
ZIP‐2 mediates dietary restriction effects in *C. elegans*. (a) Relative levels of *irg‐1* mRNA in wild‐type (N2) and *zip‐2* mutant worms on ad libitum (AL) and dietary restricted (DR) conditions at day 2 of adulthood. (b) The promoter activity of *irg‐1* in AL wild‐type (*n* = 31), DR wild‐type (*n* = 29), AL *zip‐2* mutant (*n* = 28), and DR *zip‐2* mutant worms (*n* = 28) at day 2 of adulthood. (I) P*irg‐1*::GFP expression patterns and (II) relative GFP intensity. GFP intensity of individual worms was normalized to the minimum GFP intensity value among all GFP intensity values. Scale bar: 100 μm. (c) Qualitative analysis of mitochondrial morphology in AL wild‐type (N2), DR wild‐type, AL *zip‐2* mutant worms, and DR *zip‐2* mutant worms during aging. Bars represent the proportion of worms with fragmented mitochondria. (d) MVs of wild‐type and *zip‐2* mutant worms in AL or DR conditions during aging. (e) Survival rate curves of AL wild‐type (*n* = 69), DR wild‐type (*n* = 76), AL *zip‐2* mutant worms (*n* = 70), and DR *zip‐2* mutant worms (*n* = 86). Survival data are summarized in Table S1. (f) Increase in median lifespan of DR‐treated wild‐type and *zip‐2* mutant worms compared with AL. (g) Increase in maximum lifespan of DR‐treated wild‐type and *zip‐2* mutant worms compared with AL. Relative mRNA levels were determined by RT‐qPCR, normalized to *act‐3*. Error bars represent *SEM.* ns, not significant, *p < 0.05, **p < 0.01, ****p < 0.0001; unpaired *t* test

Based on these data, we hypothesized that ZIP‐2 activation contributes to DR‐induced longevity in *C. elegans,* downstream of translational inhibition. To test this hypothesis, first we examined ZIP‐2 activity in response to DR in *C. elegans* (see Methods for DR regimen). We found that the ZIP‐2 target gene *irg‐1* was more highly expressed in wild‐type strains (N2) fed a DR regimen compared with those fed ad libitum (AL) (Figure 1a). Similarly, P*irg‐1*::GFP transgenic worms fed a DR regimen showed higher expression of the ZIP‐2 activation reporter (GFP) than those fed AL (Figure 1b). Both of these DR‐mediated effects required ZIP‐2 (Figure 1a, b). The *C. elegans* feeding‐defective mutant *eat‐2*, which mimics DR (Lakowski & Hekimi, 1998), also showed higher *irg‐1* expression than wild‐type strains, and this also required ZIP‐2 (Figure S1a, b). Thus, reduced caloric intake activates ZIP‐2.

Dietary restriction is known to extend longevity in several model organisms and to improve metabolic health in humans (Lopez‐Lluch et al., 2006; Martin et al., 2016). To test whether ZIP‐2 mediates the beneficial effects of DR, we evaluated the consequences of a *zip‐2* mutation on mitochondrial integrity, physical ability, and longevity of dietary restricted worms.

Mitochondria in the body wall muscle of *C. elegans* lose their tubular morphology and gradually undergo fragmentation during aging (Hahm et al., 2015); therefore, we examined how DR and *zip‐2* influence mitochondrial morphology in aged *C. elegans*. The proportion of N2 worms with fragmented mitochondria decreased by ~7‐fold when they were fed a DR regimen compared with AL at days 10–11 of adulthood (Figures 1c and S2). In contrast, DR reduced the proportion of *zip‐2* mutant worms with fragmented mitochondria by only ~2‐fold, and loss of ZIP‐2 increased the proportion of DR‐fed worms with fragmented mitochondria by ~6‐fold (Figure 1c). Therefore, we conclude that ZIP‐2 contributes to the DR‐mediated improvement in mitochondrial integrity during aging. We note that the expression level of mitochondrial fusion or fission regulating genes did not change in ZIP‐2‐ or DR‐dependent manner (Figure S3).

We recently demonstrated that *C. elegans*’ physical ability can be assessed by measuring their maximum velocity (MV) (Hahm et al., 2015) and modeled after the short physical performance battery test (SPPB) for humans (Guralnik et al., 1994). Therefore, to examine whether *zip‐2* influences the decline in physical ability during aging, we monitored MV. At the early adult stage, all tested worms showed a similar maximum velocity (MV); however, at day 10 of adulthood, *zip‐2* mutant worms fed DR exhibited a significantly lower MV (0.17 mm/s) than wild‐type worms fed DR (0.24 mm/s) (Figure 1d). Thus, loss of *zip‐2* accelerates the decline in physical ability during aging under DR conditions. Together, our findings reveal that ZIP‐2 contributes to the DR‐mediated increase in physical ability and mitochondrial integrity in aged worms, consistent with previous observations that reduced MV correlates with decreased mitochondrial integrity during aging in *C. elegans* (Hahm et al., 2015).

Next, we investigated the role of *zip‐2* in the DR‐mediated extension of longevity in *C. elegans* (Greer & Brunet, 2009). We found that DR‐fed *zip‐2* mutant worms showed substantially diminished median and maximum lifespans compared with DR‐fed N2 worms (Figure 1e and Table S1). Importantly, loss of *zip‐2* reduced the DR‐induced extension of median lifespan by half (Figure 1f) and almost completely eliminated the DR‐induced extension of maximum lifespan (Figure 1g). In addition, the lifespan of *eat‐2* mutants is significantly reduced by RNAi‐mediated depletion of *zip‐2* (Figure S4, Table S1). Further, we found that the extension of lifespan induced by other nutritional interventions, including dietary deprivation (DD) (Kaeberlein et al., 2006; Lee et al., 2006) or dilution peptone (DP) (Hosono, Nishimoto, & Kuno, 1989), was significantly decreased in *zip‐2* mutant worms compared with N2 (Figure S5, Table S1). Together, these findings suggest that ZIP‐2 contributes to multiple nutritional intervention mechanisms that extend lifespan in *C. elegans*.

**Figure 2:**
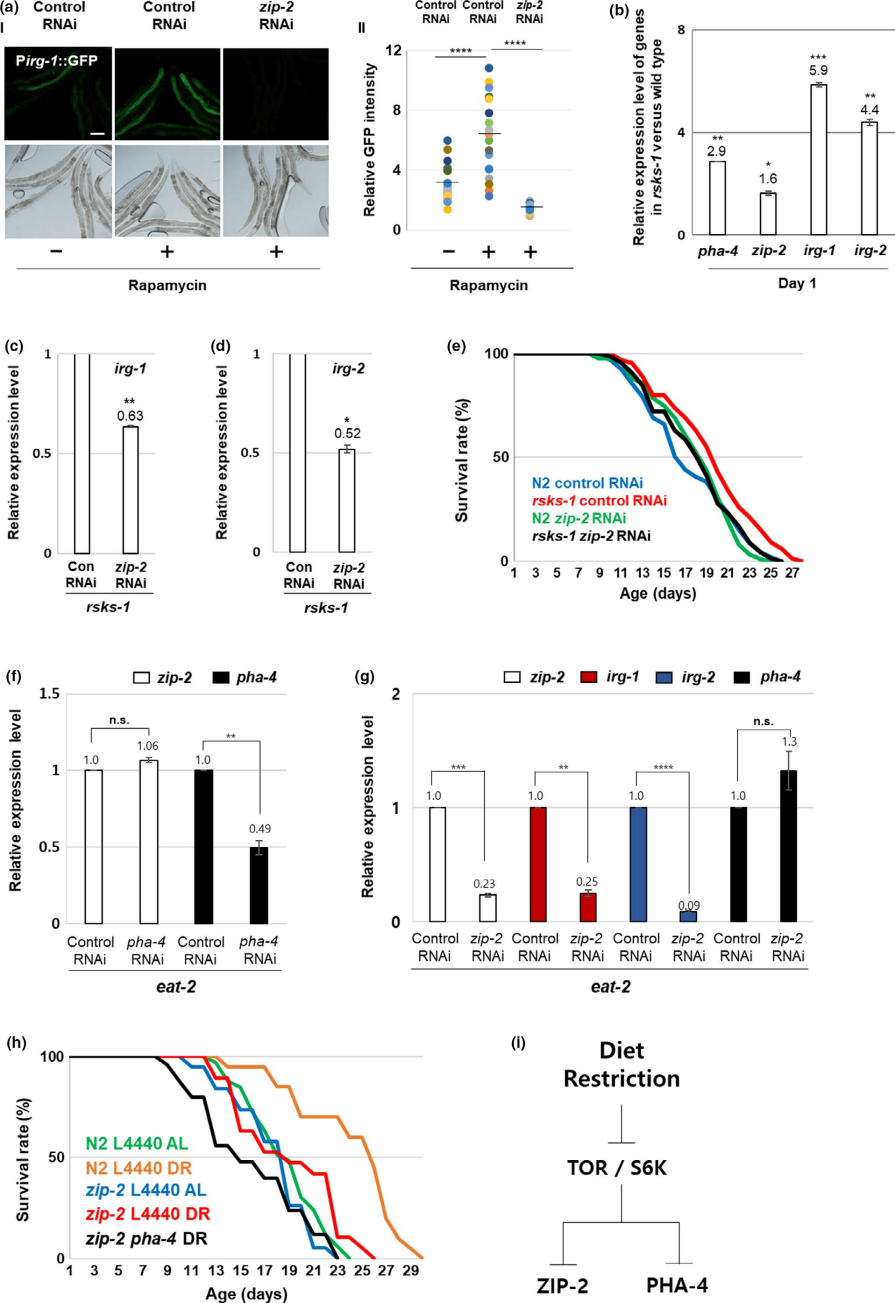
ZIP‐2 activity is increased by inhibition of TOR/S6K pathway. (a) (I) P*irg‐1*::GFP expression patterns with (*n* = 19) or without (*n* = 19) rapamycin in control RNAi and *zip‐2* RNAi worms with rapamycin (*n* = 19). Scale bar: 100 μm. (II) Relative GFP intensity. GFP intensity of individual worms was normalized to the minimum GFP intensity value among all GFP intensity values. (b) Relative transcript levels of *pha‐4*, *zip‐2*, *irg‐1*, and *irg‐2* in *rsks‐1* mutant worms compared with wild‐type strains. (c, d) Relative expression levels of *irg‐1* (c) and *irg‐2* (d) in *rsks‐1* mutant worms treated with control RNAi or *zip‐2* RNAi. (e) Survival rate curves of wild‐type in control RNAi (*n* = 91), wild‐type in *zip‐2* RNAi (*n* = 84), *rsks‐1* mutant worms in control RNAi (*n* = 65), and *rsks‐1* mutant worms in *zip‐2* RNAi (*n* = 65). Survival data are summarized in Table S1. (f) Relative expression levels of *zip‐2* and *pha‐4* in *eat‐2* mutant worms treated with control RNAi or *pha‐4* RNAi. (g) Relative expression levels of *zip‐2*, *irg‐1*, *irg‐2*, and *pha‐4* in *eat‐2* mutant worms treated with control RNAi or *zip‐2* RNAi. (h) Survival rate curves of wild‐type (N2) in control RNAi AL (*n* = 33), wild‐type in control RNAi DR (*n* = 40), *zip‐2* mutant worms in control RNAi AL (*n* = 38) and in control RNAi DR (*n* = 38), and in *pha‐4* RNAi DR (*n* = 50). Survival data are summarized in Table S1. (i) A schematic diagram for the ZIP‐2 activation in DR condition. Relative expression levels were determined by RT‐qPCR. All tested gene levels were normalized to *act‐3*. Error bars represent *SEM.* ns, not significant, *p < 0.05, **p < 0.01, ***p < 0.001, ****p < 0.0001; unpaired *t* test

Dietary restriction extends the lifespan of *C. elegans* by inhibiting the “target of rapamycin” (TOR) nutrient signaling pathway (Hansen et al., 2007). To determine whether TOR inhibition is sufficient to increase ZIP‐2 activity in *C. elegans*, we treated worms with the TOR antagonist rapamycin. Rapamycin (100 µM) treatment resulted in increased expression of the ZIP‐2 activation reporter (GFP) in control P*irg‐1*::GFP reporter worms but not in *zip‐2* RNAi reporter worms (Figure 2a). These data suggest that ZIP‐2 is activated by TOR inhibition in *C. elegans*.

S6 kinase (S6K), a key regulator of mRNA translation, is a substrate of TOR and downstream effector of the TOR pathway. Under favorable conditions, S6K functions as a positive mediator of the TOR pathway to regulate cellular and organismal growth (Montagne et al., 1999). Rapamycin indirectly inhibits S6K activity (Choo, Yoon, Kim, Roux, & Blenis, 2008), and direct inhibition of S6K extends lifespan in multiple organisms, including *C. elegans* (Hansen et al., 2007; Kapahi et al., 2010). The *C. elegans* homolog of S6K is *rsks‐1*, and we found that *rsks‐1* mutants had elevated expression of the ZIP‐2 target genes *irg‐1* and *irg‐2*, as well as *zip‐2* itself, compared with wild‐type worms (Figure 2b). The forkhead box transcription factor *pha‐4* is a downstream effector of TOR/S6K, and *pha‐4* expression was also elevated in *rsks‐1* worms compared with wild‐type worms, as expected (Figure 2b) (Sheaffer, Updike, & Mango, 2008). RNAi‐mediated depletion of *zip‐2* resulted in reduced expression of *irg‐1* and *irg‐2* in *rsks‐1* mutant worms (Figure 2c, d), and the extension of median lifespan by the *rsks‐1* mutation was significantly diminished by *zip‐2* RNAi (Figure 2e and Table S1). Thus, reduced TOR/S6K signaling leads to elevated ZIP‐2 activity, consistent with the increase in ZIP‐2 activity mediated by translational inhibition (Dunbar et al., 2012), and ZIP‐2 is necessary for the lifespan extension mediated by S6K inhibition.

PHA‐4 was previously shown to regulate lifespan extension downstream of S6K inhibition (Sheaffer et al., 2008), the *eat‐2* mutation (Panowski, Wolff, Aguilaniu, Durieux, & Dillin, 2007), and DR (Panowski et al., 2007). Thus, like ZIP‐2, PHA‐4 is a key downstream regulator of DR via the TOR/S6K signaling pathway. Both *zip‐2* and *pha‐4* showed increased expression in *eat‐2* mutants relative to wild‐type worms (Figure S6) (Panowski et al., 2007). To examine the relationship between *pha‐4* and *zip‐2*, we used RNAi to reduce the expression of each gene in an *eat‐2* mutant background. We found that *pha‐4* RNAi did not affect the expression of *zip‐2* (Figure 2f), nor did *zip‐2* RNAi affect the expression of *pha‐4* (Figure 2g). Furthermore, we found that the median lifespan of *zip‐2* mutant strains was significantly decreased compared with wild‐type in DR condition (*p* < 0.0001) (Figure 2h and Table S1). However, the median lifespan of *zip‐2* fed *pha‐4* RNAi DR was significantly shorter than the median lifespan of *zip‐2* fed L4440 RNAi DR (*p* < 0.001) (Figure 2h and Table S1). These data suggest that DR‐mediated inhibition of TOR/S6K activates two parallel pathways involving the ZIP‐2 and PHA‐4 transcription factors (Figure 2i), and that ZIP‐2 and PHA‐4 independently regulate longevity in DR‐fed *C. elegans*.

Mutations in DAF‐2, the insulin‐like growth factor 1 receptor, mimic a low nutritional status (Kimura, Riddle, & Ruvkun, 2011) and increase longevity in *C. elegans*. We hypothesized that *zip‐2* contributes to the extension of lifespan by *daf‐2* mutations, similar to DR. However, we found that the ZIP‐2 activation reporter P*irg‐1*::GFP showed similar levels of GFP expression in *daf‐2* mutant and wild‐type strains at day 1 of adulthood (Figure S7a). Furthermore, we confirmed that *daf‐2* mutation fully extends lifespan in *zip‐2* RNAi condition (Figure S7b and Table S1). Thus, ZIP‐2 and DAF‐2 act independently to control longevity in *C. elegans*.

In summary, we found that ZIP‐2, an innate immune signal in *C. elegans* (Estes, Dunbar, Powell, Ausubel, & Troemel, 2010), is activated in DR by inhibition of the TOR/S6K pathway. ZIP‐2 acts independently of PHA‐4 and DAF‐2 in extending lifespan. Our results are consistent with a recent report showing that DR activates innate immune functions in *Drosophila* via TOR inhibition (Lee, Rayyan, Liao, Edery, & Pletcher, 2017), implying that the molecular pathways that link DR and innate immunity appear to be conserved in worms and flies. We further found that DR‐activated ZIP‐2 improves longevity as well as health parameters such as mitochondrial integrity and physical ability in aging *C. elegans*, suggesting that the positive effects on health and longevity induced by DR are due in part to the activation of innate immunity. Thus, we argue that increased immunity through the practice of DR is important to increased longevity and healthy aging. Furthermore, we propose that over‐feeding may suppress innate immunity, thereby accelerating aging.

## CONFLICT OF INTEREST

None declared.

## AUTHOR CONTRIBUTIONS

J.H.H. and H.G.N. conceived and designed the study and wrote the manuscript. J.H.H. and C.L.J. performed the experimental works and analyzed the data. J.H.H. and H.G.N. edited the manuscript.
