# Supplementary material for: Diet restriction‐induced healthy aging is mediated through the immune signaling component ZIP‐2 in Caenorhabditis elegans
Source: Aging Cell. 2019 Jun 18;18(5):e12982. doi: 10.1111/acel.12982 (PMC6718572; doi:10.1111/acel.12982)
Supplement: Supplementary file 1 [file ACEL-18-e12982-s001.docx]

**Diet restriction-induced healthy aging is mediated through the immune signaling component ZIP-2 in *C. elegans***

Jeong-Hoon Hahm^1*^, ChoLong Jeong^1^, Hong Gil Nam^1,2*^

^1^Center for Plant Aging Research, Institute for Basic Science, Daegu, 42988, Republic of Korea

^2^Department of New Biology, DGIST, Daegu, 42988, Republic of Korea

*Correspondence: [hahmjh0505@ibs.re.kr](mailto:hahmjh0505@ibs.re.kr) (J.H.H), [nam@dgist.ac.kr](mailto:nam@dgist.ac.kr) (H.G.N)

**
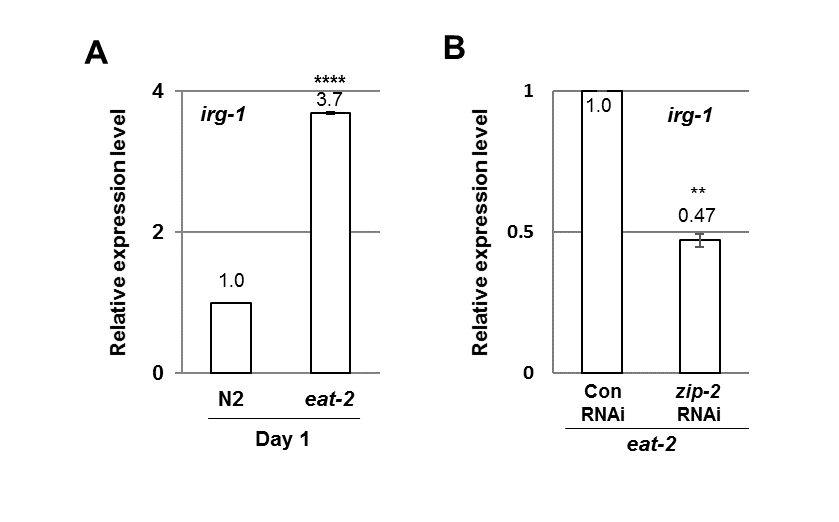
Fig. S1** ZIP-2 activity increases in *eat-2* mutants.

(A) Relative levels of *irg-1* mRNA in wild-type (N2) and *eat-2* mutants at day 1 of adulthood. (B) Relative levels of *irg-1* mRNA in *eat-2* mutant worms with control RNAi or *zip-2* RNAi. Relative mRNA levels were determined by RT-qPCR, normalized to *act-3*. Error bars represent SEM. ** P < 0.01, **** P < 0.0001; unpaired *t*-test.

**Fig. S2** ZIP-2 mediates the improvement of mitochondrial integrity in aging by dietary restriction (DR). **
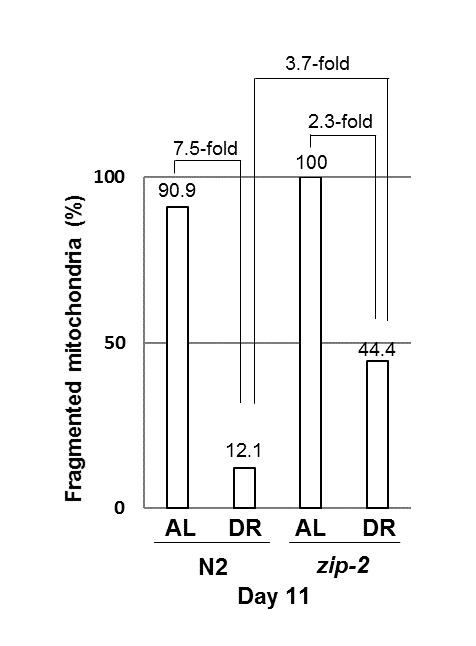
**

Qualitative analysis of mitochondrial morphology in AL wild-type (N2) (n=42), DR wild-type (n=52), AL *zip-2* mutant worms (n=42), and DR *zip-2* mutant worms (n=46) at day 11 of adulthood. Bars represent the proportion of worms with fragmented mitochondria.

**Fig. S3** Expression level of mitochondrial fusion or fission genes in dietary restriction (DR).

**
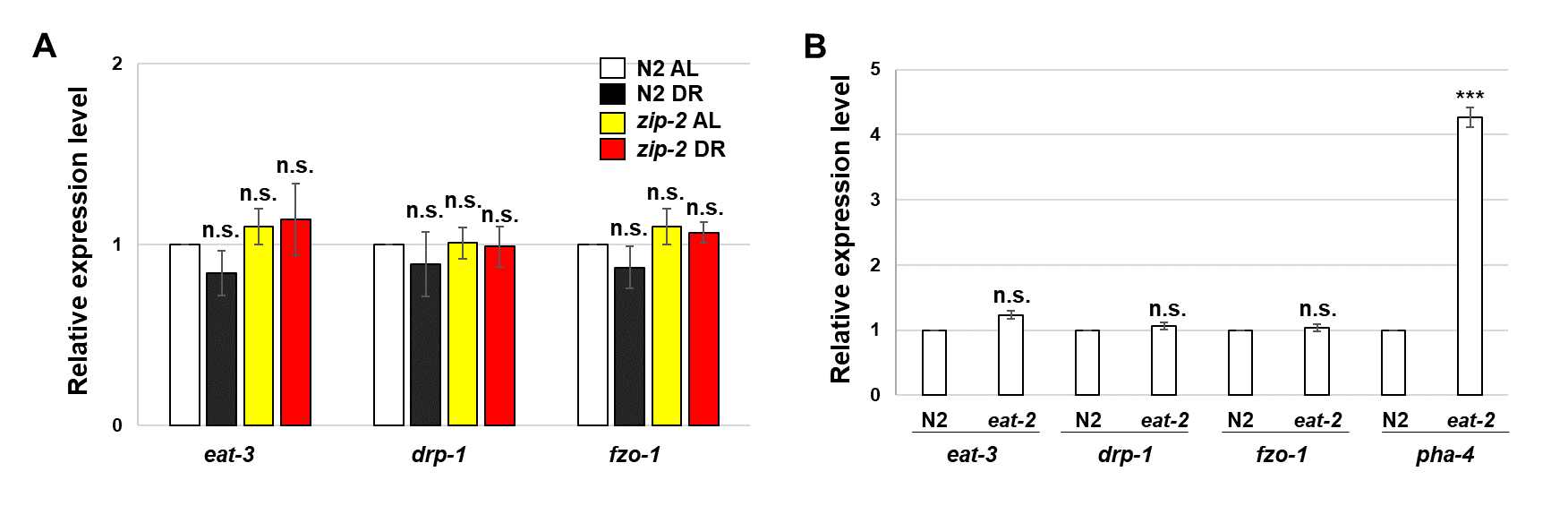
**

(A) Expression level of mitochondrial fusion (*eat-3* and *fzo-1*) or fission (*drp-1*) genes in wild-type (N2), *zip-2* mutant stains in AL or DR conditions. (B) Expression level of mitochondrial fusion (*eat-3* and *fzo-1*) or fission (*drp-1*) genes in N2 and *eat-2* mutant strains. Increased expression of *pha-4* in *eat-2* mutants served as a positive control (Panowski, Wolff, Aguilaniu, Durieux, & Dillin, 2007). Relative mRNA levels were determined by RT-qPCR, normalized to *act-3*. Error bars represent SEM. n.s.= not significant, *** P < 0.001; unpaired *t*-test.

**Fig. S4** ZIP-2 mediates the extension of lifespan in *eat-2* mutants.


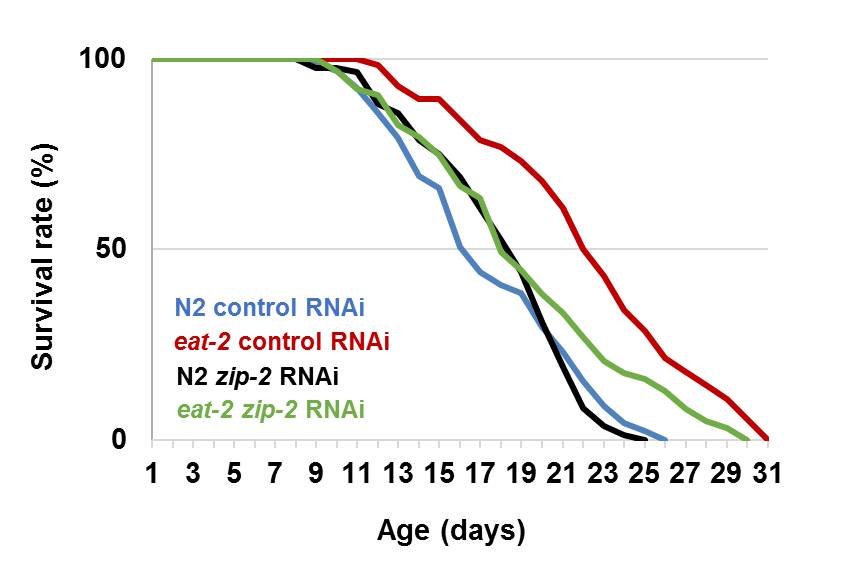


Survival rate of wild-type strains (N2) with control RNAi (n=91) or *zip-2* RNAi (n=84) and *eat-2* mutant worms with control RNAi (n=56) or *zip-2* RNAi (n=63). Survival data are summarized in Table S1.

**Fig. S5** ZIP-2 mediates the extension of lifespan by dietary deprivation (DD) or dilution peptone (DP).


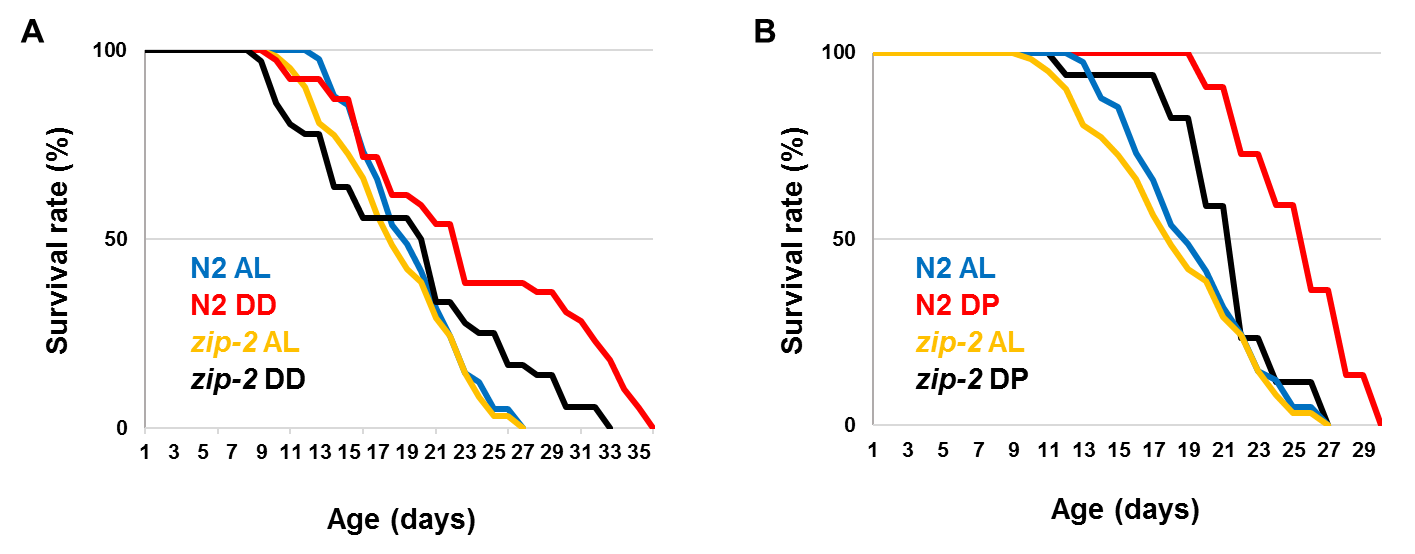


Survival rate of wild-type strains (N2) or *zip-2* mutant worms in AL or DD or DP conditions. Survival data are summarized in Table S1.

**Fig. S6** Transcript levels of *zip-2* and *pha-4* in *eat-2* mutation.


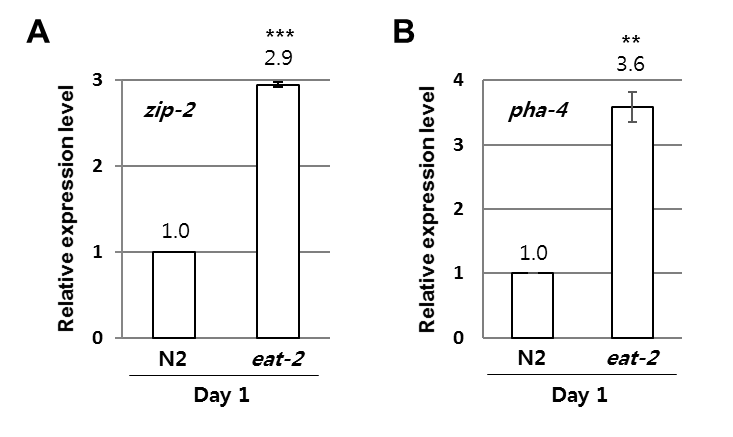


(A and B) Relative expression level of *zip-2* (A) and *pha-4* (B) in wild-type (N2) and *eat-2* mutant worms at day 1 of adulthood. Relative expression levels were determined by RT-qPCR, normalized to *act-3*. Error bars represent SEM. ** P < 0.01, *** P < 0.001; unpaired *t*-test.

**
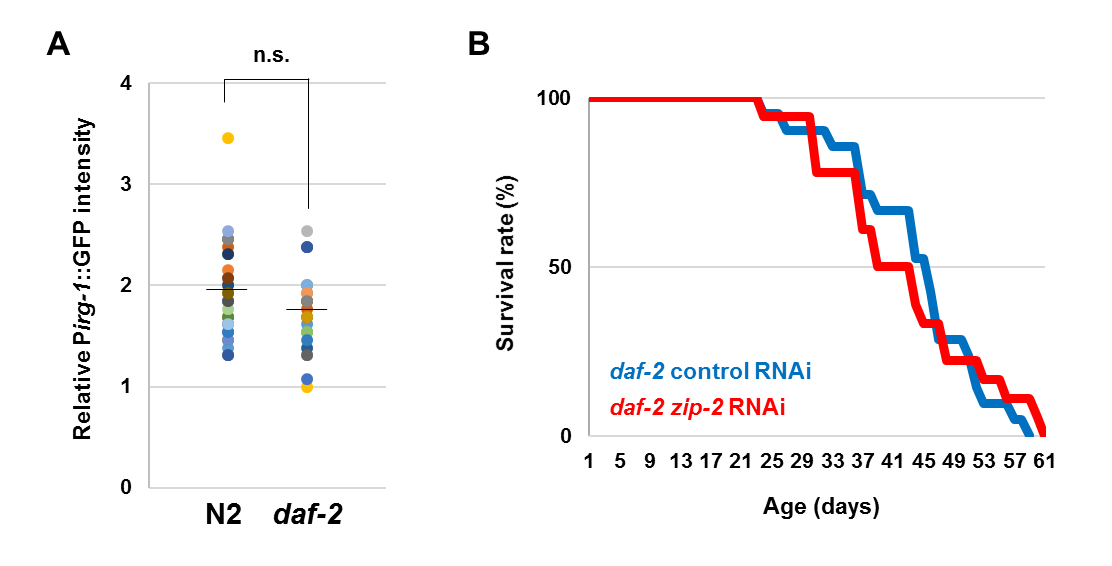
Fig. S7** ZIP-2 acts independently of DAF-2

(A) Relative P*irg-1*::GFP intensity in day 1 of adulthood wild-type (N2) (n=35) and *daf-2* mutant strains (n=23). GFP intensity of individual worms was normalized to the minimum GFP intensity value among all GFP intensity values. (B) Survival rate of *daf-2* mutant strains in control or *zip-2* RNAi. Survival data are summarized in Table S1. n.s.= not significant; unpaired *t*-test.

| **Strain** | **Dietary condition** | **RNAi**  **Treatment** | **Median Lifespan (±SEM days)** | **Sample size/trials** | **P-value** | **Related Figure** |
| --- | --- | --- | --- | --- | --- | --- |
| N2 | AL **(a)** | - | 17.71±0.4 | 69/2 |  | Fig. 1E |
|  | DR | - | 22.14±0.44 | 76/2 | *P*<0.0001 **(a)** |  |
| *zip-2* | AL | - | 16.46±0.45 | 70/2 | *P*=0.12 **(a)** |  |
|  | DR | - | 18.78±0.35 | 86/2 | *P*=0.07 **(a)** |  |
| N2 | AL **(b)** | L4440 RNAi | 17.45±0.45 | 91/2 |  | Fig. 2E |
|  | AL | *zip-2* RNAi | 18.08±0.41 | 84/2 | *P*=0.89 **(b)** |  |
| *rsks-1* | AL | L4440 RNAi | 19.60±0.54 | 65/2 | *P*<0.01 **(b)** |  |
|  | AL | *zip-2* RNAi | 18.11±0.50 | 65/2 | *P*=0.61 **(b)** |  |
| N2 | AL | L4440 RNAi | 18.79±0.53 | 33/1 | *P*=0.08 **(c)** | Fig. 2H |
|  | DR | L4440 RNAi | 24.30±0.68 | 40/1 | *P*<0.0001 **(c)** |  |
| *zip-2* | AL | L4440 RNAi | 17.84±0.51 | 38/1 | *P*<0.05 **(c)** |  |
|  | DR **(c)** | L4440 RNAi | 19.16±0.69 | 38/1 |  |  |
|  | DR | *pha-4* RNAi | 16.04±0.62 | 50/1 | *P*<0.001 **(c)** |  |
| N2 | AL | L4440 RNAi | 17.45±0.45 | 91/2 | *P*<0.0001 **(d)** | Fig. S4 |
|  | AL | *zip-2* RNAi | 18.08±0.41 | 84/2 | *P*<0.0001 **(d)** |  |
| *eat-2* | AL **(d)** | L4440 RNAi | 22.36±0.68 | 56/2 |  |  |
|  | AL | *zip-2* RNAi | 19.21±0.66 | 63/2 | *P*<0.0001 **(d)** |  |
| N2 | AL **(e)** | - | 19.46±0.59 | 41/1 |  | Fig. S5A |
|  | DD | - | 23.62±1.30 | 39/1 | *P*<0.001 **(e)** |  |
| *zip-2* | AL **(f)** | - | 18.48±0.56 | 62/1 | *P*=0.47 **(e)** |  |
|  | DD | - | 19.42±1.18 | 36/1 | *P*=0.19 **(e)**  *P*=0.06 **(f)** |  |
| N2 | AL **(g)** | - | 18.35±0.46 | 46/1 |  | Fig. S5B |
|  | DP **(h)** | *-* | 25.45±0.47 | 44/1 | *P*<0.0001 **(g)** |  |
| *zip-2* | AL | *-* | 18.73±0.77 | 30/1 | *P*=0.18 **(g)** |  |
|  | DP | *-* | 21.29±0.58 | 34/1 | *P*<0.001 **(g)**  *P*<0.0001 **(h)** |  |
| *daf-2* | AL **(i)** | L4440 RNAi | 43.95±1.38 | 42/1 |  | Fig. S7B |
|  | AL | *zip-2* RNAi | 42.50±1.70 | 36/1 | *P*=0.77 **(i)** |  |

**Table S1** Median lifespans of *C. elegans* cohorts*.*

Notes: Each (alphabet) mark on the P value represents a P value that matches the each dietary restriction condition (alphabet).

**References**

Panowski, S. H., Wolff, S., Aguilaniu, H., Durieux, J., & Dillin, A. (2007). PHA-4/Foxa mediates diet-restriction-induced longevity of C-elegans. *Nature, 447*(7144), 550-+. doi:10.1038/nature05837
