# Supplementary material for: Diet restriction‐induced healthy aging is mediated through the immune signaling component ZIP‐2 in Caenorhabditis elegans
Source: Aging Cell. 2019 Jun 18;18(5):e12982. doi: 10.1111/acel.12982 (PMC6718572; doi:10.1111/acel.12982)
Supplement: Supplementary file 3 [file ACEL-18-e12982-s003.docx]

**Materials and methods**

**Strains**

All strains were maintained at 20℃. The following strains were used in this study. N2 wild-type, *zip-2(ok3730)*, *eat-2(ad1116)*, AU133 agIs17 [*myo-2*p::mCherry + *irg-1*p::GFP] IV, PD4251 ccIs4251 [(pSAK2) *myo-3*p::GFP::LacZ::NLS + (pSAK4) *myo-3*p::mitochondrial GFP + *dpy-20*(+)]; *dyp-20(e1282)*, *zip-2(ok3730)*; agIs17 [myo-2p::mCherry + irg-1p::GFP] IV, *zip-2(ok3730)*; ccIs4251 [(pSAK2) myo-3p::GFP::LacZ::NLS + (pSAK4) myo-3p::mitochondrial GFP + dpy-20(+)], *daf-2(e1370);* agIs17 [myo-2p::mCherry + irg-1p::GFP] IV, and *rsks-1(tm1714)*.

**Dietary restriction experiments**

For the DR condition, the amount of bacteria on the nematode growth medium (NGM) plate was reduced by limiting the density of *E. coli* and removing peptone (Hahm, Kim, & Paik, 2011; Hosono, Nishimoto, & Kuno, 1989). The density of bacteria on plates was defined as the value of optical density (OD) measured by an EPOCH^2^ (BioTek). The OD value of bacteria in the DR condition is 0.36. For DR-related experiments, synchronized worms grown on the AL plate were transferred to AL or DR plates at the young adult stage. After transferring, the worms were used for the following experiments: 48 hours after transfer, worms were used for RT-qPCR experiments (Fig. 1A), to observe the activity of the *irg-1* promoter (Fig. 1B), and to measure mitochondrial integrity, physical ability, and life span (Fig. 1C-G).

The dilution peptone (DP) lifespan assay was performed as in (Hosono et al., 1989). Synchronized worms were obtained by placing adult worms on assay plates (0.0025 g/L peptone with 0.36 OD value of bacteria) and removing the worms after 6 h. The number of live animals was scored every day until death.

The dietary deprivation (DD) lifespan assay was performed as in (Kaeberlein et al., 2006; Lee et al., 2006), with modifications. Adult worms laid eggs on NGM containing heat-killed OP50. When progeny grew to the L4 stage, worms were transferred to NGM containing heat-killed OP50 supplemented with 0.1 mg/ml 5-flurodeoxyuridine (FUDR). At day 2 of adulthood, worms were transferred to either NGM containing heat-killed OP50 with FUDR (AL condition) or NGM with only FUDR (DD condition). The number of live animals was scored every day until death.

Table. S2. Methods of dietary restriction in *C. elegans*

| Method | DR (In this study) | DP | DD |
| --- | --- | --- | --- |
| Medium source of Food | *E. coli* (0.36 OD) | *E. coli* (0.36 OD) | No *E. coli* |
| Peptone treatment | No | 0.0025 g/L | 2.5 g/L |
| Timing of DR treatment | Young adult stage | Birth | Day 2 of adulthood |
| Percentage of lifespan extension in wild-type strains (%) | 25.0% | 38.7% | 21.4% |

**Quantitative-RT PCR**

Total RNA was extracted by using miRNeasy mini kit (Qiagen, Cat No. 217004). cDNA was generated by using a reverse transcription system (ImProm-II, Promega, Cat No. A3800) and was used for quantitative PCR. Quantitative real time PCR was performed with SYBR green dye (TOPreal™ qPCR 2X PreMIX, Enzynomics, Cat No. RT500) using CFX96^TM^ Real-time C1000 Touch Thermal cycler (Bio-Rad) and analyzed using ΔΔCt methods described in the manufacturer’s manual. Sequences of primers used for quantitative RT-PCR analysis; *zip-2*-Forward: GTTCTTTCCACAGCTTGTGC, *zip-2*-Reverse: GATGACGAATCGGACGATAC, *irg-1*-Forward: GCTGAAATTCACTTGTAGTGAG, *irg-1*-Reverse: GAGACCATAATTTCAATTGCTC, *irg-2*-Forward: CACCTCATTATTGCATTGTTTC, *irg-2*-Reverse: GTTGTAGACTTTTGAAAGGTTG, *pha-4*-Forward: CTGTTAATCACAGTCAACCTAC, *pha-4*-Reverse: GTGTTGTTCAGGAAATTCTGG, *act-3*-Forward: AAGTCATCACCGTCGGAAAC, and *act-3*-Reverse: TTCCTGGGTACATGGTGGTT.

**Life span analysis**

Life span was assessed on NGM plates at 20 °C. The number of live animals was scored every day until death. Life span was analyzed by Oasis survival analysis software (Yang et al., 2011).

**Qualitative analysis of mitochondrial morphology**

Mitochondrial morphology was examined in PD4251 strain, and morphological categories were defined as previously described (Hahm et al., 2015). Imaging was obtained using a microscope equipped with a C-Apochromat 40x/1.20W Korr FCS M27 and a photo-multiplier tube (PMT). Fluorescent z stacks of individual animals (1 μm/slice) was acquired using Zen 2011software (black edition). For imaging, worms were immobilized using 100 mM sodium azide.

**Measurement of worm’s maximum velocity (MV)**

MV measurement was performed as previously described (Hahm et al., 2015).

**RNAi experiments**

For RNAi experiments, we used commercial C. elegans RNAi feeding libraries generated by the Ahringer laboratory (Geneservice Ltd., Cambridge, UK). RNA interference *Escherichia coli* strains were cultured as previously described (Kamath, Martinez-Campos, Zipperlen, Fraser, & Ahringer, 2001). For Fig. 2A, adult worms were transferred to each RNAi plate and their progeny examined. For Fig. 2C-2F, RNAi treatment was for 72 hours from young adulthood. For Fig. S2, adult worms were transferred to each RNAi plate and their progeny examined for life span.

**Rapamycin treatment experiments:**

Worms were treated with rapamycin as described previously (Robida-Stubbs et al., 2012) for 48 hours at young adult stage. Rapamycin (LC laboratories: R-5000) was dissolved in DMSO at 50 mg/ml. The final concentration of rapamycin was 100 µM. P*irg-1*::GFP expression was observed using an Eclipse Ni (Nikon). Control plates contained an appropriate DMSO concentration.

**References**

Hahm, J.-H., Kim, S., DiLoreto, R., Shi, C., Lee, S.-J. V., Murphy, C. T., & Nam, H. G. (2015). C. elegans maximum velocity correlates with healthspan and is maintained in worms with an insulin receptor mutation. *Nature Communications, 6*. doi:10.1038/ncomms9919

Hahm, J.-H., Kim, S., & Paik, Y.-K. (2011). GPA-9 is a novel regulator of innate immunity against Escherichia coli foods in adult Caenorhabditis elegans. *Aging Cell, 10*(2), 208-219. doi:10.1111/j.1474-9726.2010.00655.x

Hosono, R., Nishimoto, S., & Kuno, S. (1989). ALTERATIONS OF LIFE-SPAN IN THE NEMATODE CAENORHABDITIS-ELEGANS UNDER MONOXENIC CULTURE CONDITIONS. *Experimental Gerontology, 24*(3), 251-264. doi:10.1016/0531-5565(89)90016-8

Kaeberlein, T. L., Smith, E. D., Tsuchiya, M., Welton, K. L., Thomas, J. H., Fields, S., . . . Kaeberlein, M. (2006). Lifespan extension in Caenorhabditis elegans by complete removal of food. *Aging Cell, 5*(6), 487-494. doi:10.1111/j.1474-9726.2006.00238.x

Kamath, R. S., Martinez-Campos, M., Zipperlen, P., Fraser, A. G., & Ahringer, J. (2001). Effectiveness of specific RNA-mediated interference through ingested double-stranded RNA in Caenorhabditis elegans. *Genome Biol, 2*(1), Research0002. doi:10.1186/gb-2000-2-1-research0002

Lee, G. D., Wilson, M. A., Zhu, M., Wolkow, C. A., de Cabo, R., Ingram, D. K., & Zou, S. (2006). Dietary deprivation extends lifespan in Caenorhabditis elegans. *Aging Cell, 5*(6), 515-524. doi:10.1111/j.1474-9726.2006.00241.x

Robida-Stubbs, S., Glover-Cutter, K., Lamming, D. W., Mizunuma, M., Narasimhan, S. D., Neumann-Haefelin, E., . . . Blackwell, T. K. (2012). TOR signaling and rapamycin influence longevity by regulating SKN-1/Nrf and DAF-16/FoxO. *Cell Metab, 15*(5), 713-724. doi:10.1016/j.cmet.2012.04.007

Yang, J. S., Nam, H. J., Seo, M., Han, S. K., Choi, Y., Nam, H. G., . . . Kim, S. (2011). OASIS: online application for the survival analysis of lifespan assays performed in aging research. *PLoS One, 6*(8), e23525. doi:10.1371/journal.pone.0023525
