# Supplementary material for: Diet restriction‐induced healthy aging is mediated through the immune signaling component ZIP‐2 in Caenorhabditis elegans
Source: Aging Cell. 2019 Jun 18;18(5):e12982. doi: 10.1111/acel.12982 (PMC6718572; doi:10.1111/acel.12982)
Supplement: Supplementary file 2 [file ACEL-18-e12982-s002.docx]

**Supervising Editor's Comments to Author:** 
Both reviewers agree that the manuscript is interesting. However, they have a number of concerns and request either minor or major revisions which must be addressed before the study can be re-considered for publications. In any resubmission the authors should provide point by point responses.

We provide the point-by-point responses below.

**Reviewers' Comments to Author:**

**Reviewer: 1** 
Hahm and co-authors explored the role of ZIP-2, a bZIP transcription factor that induces innate immune responses, in lifespan and healthspan in C elegans. The study spawned from the factor that dietary restriction (DR) enhances the worm’s defense against bacterial infection and it is a well-established, if not the only, intervention that consistently promote longevity in various model organisms. Consistent with findings by others the study showed that translational inhibition via the TOR/rsks-1 pathway regulates the ZIP-1 activity (and to a small extend, the expression) and the consequent immune response as assayed by irg-1 expression level.

This work could be interesting to the Aging Cell readership that interested in the molecular mechanism(s) underlying the longevity promoting effects of DR. The author demonstrated that the beneficial effect of ZIP-2 under the control of rsks-1 is independent of daf-2 pathway, adding a new piece in the synergistic lifespan regulation by rsks-1 and daf-2 pathways (Chen, 2013, Cell Reports). A novelty of the study is that the authors also identified ZIP-2 as a regulator of the mitochondrial morphology change (which is associated mitochondrial function and aging) and the worm’s healthspan using their mobility assay. 

**Query 1-1.** There are certain limitations in the study. The work is quite descriptive and the connection linking ZIP-2 to mitochondrial morphology/function and to mobility was not examined. For example, the authors could test the potential regulation of mitochondrial fusion and fission processes by ZIP-2.

**Response 1-1.** We thank the reviewer for this suggestion. Mitochondrial quality is controlled by continuous fusion and fission (Kowald & Kirkwood, 2011), which in turn are regulated by dynamin proteins. In mammals, the fusion of the mitochondrial outer membrane is mediated by mitofusin (MFN) 1 and 2 and inner mitochondrial membrane fusion is mediated by optic atrophy 1 (OPA1). Mitochondrial fission is mediated by dynamin related protein 1 (DRP1).

In *C. elegans*, EAT-3, FZO-1, and DRP-1 are orthologous to OPA1, MFN1, and DRP1, respectively, and play an important role in maintaining mitochondrial homeostasis (Maglioni & Ventura, 2016), and down-regulation of fusion genes or over-expression of fission gene directly leads to fragmented mitochondria in *C. elegans* (Breckenridge et al., 2008; Chaudhari & Kipreos, 2017; Jagasia, Grote, Westermann, & Conradt, 2005).

**Fig. 1.** RT-qPCR analysis of *eat-3*, *drp-1*, and *fzo-1* in N2 or *zip-2* mutant worms. Normalized to *act-3*, and to N2 AL condition.

To answer the reviewer’s concern, we investigate how ZIP-2 is linked to maintaining mitochondrial integrity in response to DR by examining the expression of *eat-3*, *drp-1* and *fzo-1* by RT-qPCR. We found that the expression of these genes was not significantly altered by DR nor by *zip-2* loss-of-function (**Fig. 1**, Fig. S3A in the revised manuscript). Further, we found that the expression of mitochondrial fusion or fission genes were similar in N2 and *eat-2* mutant strains. Increased expression of *pha-4* in *eat-2* mutants served as a positive control (Panowski, Wolff, Aguilaniu, Durieux, & Dillin, 2007) (**Fig. 2**, Fig. S3B in the revised manuscript).

**Fig. 2.** Relative expression levels of *eat-3*, *drp-1*, *fzo-1*, and *pha-4* in N2 or *eat-2* mutant worms. Normalized to *act-3*.

Together, these data suggest that the ZIP-2- and DR-dependent improvement of mitochondrial integrity is not related to the regulation of mitochondrial fusion or fission processes. We noted this in page 3, line 2-3 in the revised manuscript.

**Query 1-2**. Although the interaction between pha-4 and zip-2 was examined at the expression level, a double RNAi experiment is needed to solidly demonstrate the regulation of longevity by two parallel pathways.

**Response 1-2.** We agree with the reviewer and performed this experiment by feeding wild-type strains (N2) and *zip-2* mutant strains L4440 control RNAi or *pha-4* RNAi bacteria, in AL or DR conditions. We found that the median lifespan of *zip-2* mutant strains was significantly decreased compared to wild-type strains in DR condition (P < 0.0001). The median lifespan of wild-type L4440 RNAi was 24.3 days and *zip-2* L4440 RNAi was 19.2 days when fed DR (**Fig. 3,** Fig. 2H in the revised manuscript). However, the median lifespan of *zip-2* fed *pha-4* RNAi DR was reduced to 16.0 days, significantly shorter than the median lifespan of *zip-2* fed L4440 RNAi DR (P < 0.001). Survival data are summarized in **Table 1** and Table S1 in the revised manuscript. These data provide further support that ZIP-*2* and PHA-4 independently promote longevity of *C. elegans* fed a DR diet. We noted this in page 5, 1^st^ paragraph in the revised manuscript.

**Fig. 3.** Survival rate of N2 and *zip-2* mutant worms fed L4440 or *pha-4* RNAi bacteria in AL or DR conditions.

**Table 1.** Median lifespans of *C. elegans* cohorts*.*

Notes: (a) mark on the P value represents a P value that matches the dietary restriction condition (a).

| Strain | Dietary condition | RNAi  Treatment | Median Lifespan (±SEM days) | Sample size/trials | P-value |
| --- | --- | --- | --- | --- | --- |
| N2 | AL | L4440 RNAi | 18.79±0.53 | 33/1 | *P*=0.08 (a) |
|  | DR | L4440 RNAi | 24.30±0.68 | 40/1 | *P*<0.0001 (a) |
| *zip-2* | AL | L4440 RNAi | 17.84±0.51 | 38/1 | *P*<0.05 (a) |
|  | DR (a) | L4440 RNAi | 19.16±0.69 | 38/1 |  |
|  | DR | *pha-4* RNAi | 16.04±0.62 | 50/1 | *P*<0.001 (a) |

**Minor:**

**Query 1-3.** Page 8: “different from each other statistically”

**Response 1-3.** In modified text, the sentence was deleted.

**Query 1-4.** In the Materials and Methods, the authors should clarify the DR protocol used (extend in percentage compared to control).

**Response 1-4**. We have clarified the Materials and Methods to describe the DR method we used in this study, dilution peptone (DP) (Hosono, Nishimoto, & Kuno, 1989), and dietary deprivation (DD) (Kaeberlein et al., 2006; Lee et al., 2006). **Table 2** provides the details of the DR, DP and DD with regard to the amount of *E. coli* or peptone, timing of intervention, and percentage of lifespan extension.

**Table 2**. Nutritional interventions in this study

| Method | DR | DP | DD |
| --- | --- | --- | --- |
| *E. coli* | 0.36 OD | 0.36 OD | 0 |
| Peptone treatment | No | 0.0025 g/L | 2.5 g/L |
| Timing of intervention | Young adult stage | Birth | Day 2 of adulthood |
| Percentage of lifespan extension in wild-type strains (%) | 25.0% | 38.7% | 21.4% |

**Reviewer: 2** 
This study by Hahm et al. links activation of innate immunity to the downstream effects of dietary restriction in C. elegans. Moreover, it attributes this activation to reduced signaling through the TOR pathways. Given that similar findings have been reported in field, this study is therefore important in that it suggests conserved effects of DR across species. There are, however, some concerns with the data that should be addressed. 

**Query 2-1.** RNAi to zip-2 seems like it has adverse effects on its own in lifespan and other phenotypes such as mitochondrial integrity. The authors to a large extent handle this concern with respect to survival since the comparative effects are shown to be reduced. However, this remains a concern with mitochondrial integrity and movement, where it is hard to determine the relative effects without a broader set of timepoints across the lifespan. This is recommended for both assays, since it may rule out the notion that zip-2 mutants are defective on their own and that the effects of DR are relatively the same starting from a worse background.

**Response 2-1.** We thank the reviewer for this important point. To rule out that *zip-2* mutants are starting from a worse background, we examined mitochondrial integrity and movement of wild-type strains (N2) and *zip-2* mutant strains across their lifespans, in AL or DR conditions, as suggested.

At the early adult stage (Day 2), we did not observe fragmented mitochondria under any of the conditions, suggesting that *zip-2* mutants do not have defects in mitochondrial integrity at this stage (**Fig. 4,** Fig. 1C in the revised manuscript). At day 10, DR reduced the proportion of N2 worms with fragmented mitochondria by ~7-fold. In contrast, DR reduced the proportion of *zip-2* mutants with fragmented mitochondria by only ~2-fold at day 10 of adulthood. Further, the *zip-2* mutation increased the proportion of DR-fed worms with fragmented mitochondria by ~6-fold. From these data, we conclude that ZIP-2 contributes to the DR-mediated improvement in mitochondrial integrity during aging. We noted this in page 2, line 21-27 and page 3, line 1-2 in the revised manuscript.

**Fig. 4.** Mitochondrial morphology of N2 or *zip-2* mutant worms in AL or DR conditions.

We also observed the change of physical ability of N2 and *zip-2* mutant strains in AL or DR conditions across their lifespans. Again, at the early adult stage, all tested worms showed a similar maximum velocity (MV) (**Fig. 5,** Fig. 1D in the revised manuscript). However, by day 10 of adulthood, the MV of *zip-2* mutants fed DR (0.17 mm/s) was significantly lower than the MV of N2 fed DR (0.24 mm/s). Thus, loss of *zip-2* accelerates the decline in physical ability during aging under DR conditions. We noted this in page 3, line 4-10 in the revised manuscript.

**Fig. 5.** Maximum velocity of N2 or *zip-2* mutant worms in AL or DR conditions.

**Query 2-2.** There are many DR methods in worms and the paper could be improved by trying a couple more to demonstrate the generality of the zip-2 effect.

**Response 2-2.** We thank the reviewer for this suggested and tested whether ZIP-2 regulates longevity of *C. elegans* in other DR conditions, specifically dietary deprivation (DD) (Kaeberlein et al., 2006; Lee et al., 2006) and dilution peptone (DP) (Hosono et al., 1989). (**Fig. 6,** Fig. S5 in the revised manuscript**,** and **Table 3,** Table S1 in the revised manuscript). Please see **Table 2** and the Materials and Methods for details of these distinct nutritional interventions.

**Fig. 6.** Survival rate of N2 and *zip-2* mutant worms fed AL, DD or DP.

DD significantly increased the median lifespan of wild-type strains (N2) by 21% (P < 0.001) (the median lifespan was 19.5 days for N2 fed AL and 23.6 days for N2 fed DD). However, DD did not significantly extend the median lifespan of *zip-2* mutants (P = 0.06) (the median lifespan was 18.5 days for *zip-2* fed AL and 19.4 days for *zip-2* fed DD).

Similarly, DP significantly increased the median lifespan of N2 by 39% (P < 0.0001) (the median lifespan was 18.4 days for N2 fed AL and 25.5 days for N2 fed DP). Although DP also extended the median lifespan of *zip-2* mutants (P < 0.001), the effect was smaller (the median lifespan was 18.7 days for *zip-2* fed AL and 21.3 days for *zip-2* fed DP). Further, the extension of lifespan by DP was significantly decreased by *zip-2* loss-of-function (P < 0.0001) (the median lifespan was 25.5 days for N2 in DP and 21.3 days for *zip-2* in DP).

These data suggest that ZIP-2 is necessary for lifespan extension by various nutritional interventions. We noted this in page 3, line 20-25 in the revised manuscript.

| Strain | Dietary condition | Median Lifespan (±SEM days) | Sample size/trials | P-value | Related Figure |
| --- | --- | --- | --- | --- | --- |
| N2 | AL (a) | 19.46±0.59 | 41/1 |  | Fig. 6A |
|  | DD | 23.62±1.30 | 39/1 | *P*<0.001 (a) |  |
| *zip-2* | AL (b) | 18.48±0.56 | 62/1 | *P*=0.47 (a) |  |
|  | DD | 19.42±1.18 | 36/1 | *P*=0.19 (a)  *P*=0.06 (b) |  |
| N2 | AL (c) | 18.35±0.46 | 46/1 |  | Fig. 6B |
|  | DP (d) | 25.45±0.47 | 44/1 | *P*<0.0001 (c) |  |
| *zip-2* | AL | 18.73±0.77 | 30/1 | *P*=0.18 (c) |  |
|  | DP | 21.29±0.58 | 34/1 | *P*<0.001 (c)  *P*<0.0001 (d) |  |

**Table 3.** Median lifespans of *C. elegans* cohorts*.*

Notes: (alphabet) mark on the P value represents a P value that matches the dietary restriction condition (alphabet).

**Query 2-3.** The authors should consider looking at other longevity interventions to test the breadth of the zip-2 requirement.

**Response 2-3.** To investigate the role of ZIP-2 in other longevity intervention pathways, we examined mutations in *daf-2*, which encodes the insulin/IGF-1 receptor. *daf-2* mutations mimic a low nutrition status and double the lifespan of C. elegans (Kenyon, Chang, Gensch, Rudner, & Tabtiang, 1993).

**Fig. 7.** ZIP-2 acts independently of DAF-2.

We found that Pirg-1::GFP, the ZIP-2 activation reporter, showed similar levels of GFP expression in daf-2 mutant and wild-type strains (N2) at day 1 of adulthood (**Fig. 7A**, Fig. S7A in the revised manuscript). Further, the median lifespans of daf-2 mutants are similar in control RNAi (43.9 days) and in zip-2 RNAi (42.5 days) (P = 0.77) (**Fig. 7B**, Fig. S7B in the revised manuscript). Survival data are summarized in **Table 4** (Table S1 in the revised manuscript).

From these data, we propose that ZIP-2 acts independently of the insulin/IGF-1 signaling pathway. We noted this in page 5, 2^nd^ paragraph in the revised manuscript.

**Table 4**. Median lifespans of *C. elegans* cohorts*.*

Notes: (alphabet) mark on the P value represents a P value that matches the dietary restriction condition (alphabet).

| **Strain** | **Dietary condition** | **RNAi**  **Treatment** | **Median Lifespan (±SEM days)** | **Sample size/trials** | **P-value** |
| --- | --- | --- | --- | --- | --- |
| *daf-2* | AL (a) | L4440 RNAi | 43.95±1.38 | 42/1 |  |
|  | AL | *zip-2* RNAi | 42.50±1.70 | 36/1 | *P*=0.77 (a) |

Further, we have now extended the study and show that ZIP-2 is necessary for the lifespan extension of rsks-1 (**Fig. 8**, Fig. 2E in the revised manuscript), suggesting that ZIP-2 regulates lifespan via the TOR/RSKS-1 pathway. We noted this in page 4, line 15-19 in the revised manuscript.

Survival data are summarized in **Table 5** (Table S1 in the revised manuscript).

**Table 5.** Median lifespans of *C. elegans* cohorts*.*

**Fig. 8.** ZIP-2 mediates the lifespan extension by *rsks-1* mutation.

Notes: (alphabet) mark on the P value represents a P value that matches the dietary restriction condition (alphabet).

| **Strain** | **Dietary condition** | **RNAi**  **Treatment** | **Median Lifespan (±SEM days)** | **Sample size/trials** | **P-value** |
| --- | --- | --- | --- | --- | --- |
| N2 | AL **(a)** | L4440 RNAi | 17.45±0.45 | 91/2 |  |
|  | AL | *zip-2* RNAi | 18.08±0.41 | 84/2 | *P*=0.89 **(a)** |
| *rsks-1* | AL | L4440 RNAi | 19.60±0.54 | 65/2 | *P*<0.01 **(a)** |
|  | AL | *zip-2* RNAi | 18.11±0.50 | 65/2 | *P*=0.61 **(a)** |

**References**

Breckenridge, D. G., Kang, B. H., Kokel, D., Mitani, S., Staehelin, L. A., & Xue, D. (2008). Caenorhabditis elegans drp-1 and fis-2 regulate distinct cell-death execution pathways downstream of ced-3 and independent of ced-9. *Molecular Cell, 31*(4), 586-597. doi:10.1016/j.molcel.2008.07.015

Chaudhari, S. N., & Kipreos, E. T. (2017). Increased mitochondrial fusion allows the survival of older animals in diverse C. elegans longevity pathways. *Nat Commun, 8*(1), 182. doi:10.1038/s41467-017-00274-4

Hosono, R., Nishimoto, S., & Kuno, S. (1989). ALTERATIONS OF LIFE-SPAN IN THE NEMATODE CAENORHABDITIS-ELEGANS UNDER MONOXENIC CULTURE CONDITIONS. *Experimental Gerontology, 24*(3), 251-264. doi:10.1016/0531-5565(89)90016-8

Jagasia, R., Grote, P., Westermann, B., & Conradt, B. (2005). DRP-1-mediated mitochondrial fragmentation during EGL-1-induced cell death in C. elegans. *Nature, 433*(7027), 754-760. doi:10.1038/nature03316

Kaeberlein, T. L., Smith, E. D., Tsuchiya, M., Welton, K. L., Thomas, J. H., Fields, S., . . . Kaeberlein, M. (2006). Lifespan extension in Caenorhabditis elegans by complete removal of food. *Aging Cell, 5*(6), 487-494. doi:10.1111/j.1474-9726.2006.00238.x

Kowald, A., & Kirkwood, T. B. (2011). Evolution of the mitochondrial fusion-fission cycle and its role in aging. *Proc Natl Acad Sci U S A, 108*(25), 10237-10242. doi:10.1073/pnas.1101604108

Lee, G. D., Wilson, M. A., Zhu, M., Wolkow, C. A., de Cabo, R., Ingram, D. K., & Zou, S. (2006). Dietary deprivation extends lifespan in Caenorhabditis elegans. *Aging Cell, 5*(6), 515-524. doi:10.1111/j.1474-9726.2006.00241.x

Maglioni, S., & Ventura, N. (2016). C. elegans as a model organism for human mitochondrial associated disorders. *Mitochondrion, 30*, 117-125. doi:10.1016/j.mito.2016.02.003

Panowski, S. H., Wolff, S., Aguilaniu, H., Durieux, J., & Dillin, A. (2007). PHA-4/Foxa mediates diet-restriction-induced longevity of C-elegans. *Nature, 447*(7144), 550-+. doi:10.1038/nature05837
